# Editorial: Host-Directed Therapies for Tuberculosis

**DOI:** 10.3389/fcimb.2021.742053

**Published:** 2021-08-06

**Authors:** Diego L. Costa, Mamoudou Maiga, Selvakumar Subbian

**Affiliations:** ^1^Departmento de Bioquímica e Imunologia, Faculdade de Medicina de Ribeirão Preto, Universidade de São Paulo, Ribeirão Preto, Brazil; ^2^Programa de Pós-Graduação em Imunologia Básica e Aplicada, Faculdade de Medicina de Ribeirão Preto, Universidade de São Paulo, Ribeirão Preto, Brazil; ^3^Microbiology Department, University of Sciences, Techniques and Technologies of Bamako, Bamako, Mali; ^4^Biomedical Engineering Department, Northwestern University, Evanston, IL, United States; ^5^Public Health Research Institute Center at New Jersey Medical School, Rutgers University, Newark, NJ, United States

**Keywords:** host-directed therapy, tuberculosis, *Mycobacterium tuberculosis*, multidrug resistance, mycobacteria

Tuberculosis (TB), a highly contagious infectious disease caused by *Mycobacterium tuberculosis* (*M.tb*), is a leading cause of morbidity and mortality in humans worldwide (WHO, 2020). A major challenge for the control of TB still nowadays is the lack of a treatment regimen that can cure the disease rapidly. The standard therapy consists of at least 6 months of administration of multiple antibiotics that have side effects and therefore pose significant challenges in patient compliance. This factor, combined with an inappropriate drug prescription, contributes significantly to the emergence and spread of multidrug-resistant and extensively drug-resistant *M.tb* strains in the population, which requires more complicated treatment regimens for a much longer duration. Thus, there is an urgent need to develop novel strategies to control the global TB epidemic.

While the search for new and more effective antibacterial drugs continues, much attention has been driven towards host biological processes that contribute to TB pathogenesis and can be targeted for therapeutic interventions (Kolloli and Subbian, 2017). This emerging concept, also known as host-directed therapy (HDT), aims to optimize the host immune responses, resulting in more effective activation of the host antibacterial mechanisms combined with suppression of detrimental inflammation-driven tissue damage. This Research Topic has gathered six articles, including reviews, original articles, and a perspective contributed by well-known investigators working in specific areas of HDT in TB.

The strong inflammatory response in the lungs during TB disease is associated with a chronic granulomatous pathology that sets the stage for a long duration of antibiotic treatment because the granuloma represents a physical barrier for drug penetration (Cicchese et al., 2020). The granuloma is a result of the metabolic interplay between host immune cells and *M.tb* in order to limit the spread of the infection into the rest of the lung tissue (Kumar et al., 2019). By doing so, these inflammatory lesions also limit the contact of antibiotics with bacterial populations located in the center of the granulomas. In this Research Topic, Park et al. described the critical role of the immune metabolic cellular processes and related metabolites in TB immune response to TB infection, disease progression and severity. The authors also presented recently discovered mechanisms on how metabolic alterations during TB can lead to the development of bacterial cells that are less sensitive to anti-TB drugs (persisters) and prevent bacterial control by immune cells (Park et al.). Although more data are still needed for a better understanding of these bacterial metabolic links with the host cells, this work summarized nicely recent progress made and addressed many potential strategies that are worth studying, among which the regulation of the host cells to favor the immune metabolic pathways against TB, especially during treatment.

Autophagy, a cellular homeostasis mechanism induced during stress conditions, also enables the host cell to control intracellular pathogens, such as *M.tb* (Siqueira et al., 2018). In this Research Topic, Silwal et al. have reviewed the regulatory mechanisms of autophagy-targeting antimicrobial therapeutics against mycobacterial infection. In general, autophagy can be categorized into macro, micro, and chaperone-mediated pathways, depending on how the intracellular components (cargo) are processed for recycling. Apart from these canonical autophagy mechanisms, there are non-canonical pathways, including xenophagy, mitophagy and lipophagy involved in more selective autophagic processes. In addition to their contribution to host cell homeostasis, metabolism and recovery of cellular function after stress, these autophagy mechanisms also contribute to control intracellular *M.tb*. Several pathways such as the AMPK, mTOR/AKT, Wnt signaling as well as the transcription factors regulating the pathways, including TFEB, FOXO and NRF are attractive targets to active autophagy in host phagocytes. Thus, some of these pathways have been targeted by small molecule drugs as HDT *in vitro* and *in vivo* to induce autophagy in *M.tb*-infected host cells with the objective to improve bacterial clearance, and encouraging results have been obtained for some of these HDT drugs (Singh and Subbian, 2018).

Other HDT molecules, such as the herbal products baicalin and ajoene target inflammation and endoplasmic reticulum stress associated with autophagy. Importantly, although induction of autophagy has the potential to control intracellular bacterial growth, *M.tb* has several virulence determinants, including esx family of molecules, to defend the autophagy-mediated bactericidal activities. Yet, the mechanistic aspect of how these pathogen-derived factors influence host cell autophagy machinery is not fully understood. Nonetheless, since perturbing autophagy impacts *M.tb* survival and/or proliferation in phagocytes, it makes this host cell process an attractive HDT for TB.

Exacerbated inflammation and tissue destruction are hallmarks of progressive TB in humans (Kaufmann and Dorhoi, 2013). One mechanism that contributes to *M.tb*-mediated hyper inflammation is the NLRP3-mediated inflammasome activation (Carlsson et al., 2010). In this Research Topic, Santiago-Carvalho et al. show that signaling by the P2X7 receptor, an ATP-gated ionotropic molecule, mediates lung inflammation and tissue destruction in a murine model of experimental TB. The authors also report that among various P2X-family member genes, the transcription of P2RX7 was upregulated in the peripheral blood of patients with pulmonary TB. Similar observation was also made in the lung leukocytes of mice infected with *M.tb*. Since attenuating inflammation would favor improved clinical outcomes, the authors targeted P2X7 as HDT for TB treatment. They used a P2X7 antagonist BBG, a food additive with structural similarities to other P2X7 antagonists, to test its protective effect against active pulmonary TB in C57BL/6 mice. The authors noted a reduction in immune cell recruitment in the BBG treated, *M.tb*-infected animals compared to untreated controls. Particularly, GR1^+^ myeloid cells were prominently reduced, followed by impairment in total and CD69^+^/CD44^+^ T cell population, in the BBG-treated animals (Santiago-Carvalho et al.).

Consistent with the dampening of inflammatory response and immune cell recruitment, the BBG treated *M.tb*-infected mice showed improvement in the pathological manifestations, marked by the absence of weight loss, reduced lung bacterial load and histopathology, compared to the untreated controls. Thus, the study highlights blocking host P2X7 using small molecule inhibitors as potential way to improve the outcome of *M.tb* infection. Additional extensive studies of pharmacokinetic and pharmacodynamic nature are warranted on this class of molecules before they are evaluated in clinical trials. Since regular mice strains do not produce necrotic and cavitary granulomas, the potential of HDT for TB should be tested in animal models that produce those pathological features, which are hallmarks of active TB in humans, such as rabbits and non-human primates (Akkina et al., 2020).

Type 2 diabetes mellitus (DM2) is a common comorbidity associated with complications in TB. People living with DM2 have an increased risk of developing active TB (Jeon and Murray, 2008) and usually present more severe TB manifestations (Kumar Nathella and Babu, 2017) as well as increased treatment failure rates (Riza et al., 2014). The alterations in immune responses in diabetic people may be associated with their heightened susceptibility to *M.tb* infection (Kumar Nathella and Babu, 2017). In this Research Topic, Bobadilla-del-Valle et al. used a whole blood mycobacterial growth inhibition assay (MGIA) to compare the capacity of peripheral blood immune cells from DM2 patients (with good or poor glycemic control) and healthy individuals in controlling *M.tb* growth *in vitro*. The authors determined that blood samples from people with DM2 have impaired capacity to control bacterial growth compared to those of healthy individuals. In particular, the inhibition of bacterial growth was markedly reduced in samples from patients with poor glycemic control, which also produced lower levels of IL-6, IFNγ and TNF (Bobadilla-Del-Valle et al.). These results suggest that hyperglycemia impairs the production of cytokines by immune cells favoring bacterial replication. Additionally, this study demonstrates that the MGIA may be used as a tool to predict the ability of host immune cells in controlling *M.tb* replication that can be further used for diagnostic purposes or the design of therapeutic strategies, in particular for diabetic patients.

Besides its effects on immune cells, hyperglycemia also causes heightened systemic oxidative stress, which is common in diabetic patients (King and Loeken, 2004). Glutathione (GSH) is one of the most important antioxidant molecules in the human organism and has a crucial role in the detoxification of ROS and reactive nitrogen species (RNS) (Cooper et al., 2011). A previous study by Lagman et al. determined that individuals with DM2 have lower levels of GSH, which was associated with impaired control of *M.tb* replication *in vitro* (Lagman et al., 2015). In fact, exacerbated oxidative stress is detrimental for the host during infection with *M.tb*, resulting in enhanced inflammation, tissue destruction and bacterial dissemination (Amaral et al., 2021). In this Research Topic, To et al. evaluated whether oral supplementation with glutathione liposomes to DM2 patients could reduce oxidative stress levels and result in improved control of *M. bovis* BCG replication by macrophages isolated from these patients *in vitro*. The authors demonstrate that GSH supplementation for 3 months reduced the levels of oxidative stress in DM2 patients, as measured by decreased levels of malondialdehyde (MDA), in patients that received GSH compared to those from patients that were administered a placebo. MDA is a product of oxidative stress-driven lipid peroxidation that can be detected in plasma, peripheral blood mononuclear cells and red blood cells. Also, using a model of *in vitro* granuloma generated by DM2 patients’ peripheral blood mononuclear cells infected with *M. bovis* BCG, the authors demonstrate that granulomas containing PBMC from GSH-supplemented patients controlled bacterial replication more efficiently and produced enhanced levels of IFNγ and TNF compared to samples from placebo-treated patients (To et al.). These results suggest that GSH supplementation to DM2 patients with mycobacterial infections, such as TB, can result in more effective immune responses and control of bacterial replication, representing a potential host-directed therapy for these diseases.

In this Research Topic, Costa et al. also reviewed their previous finding on the role of host-directed therapy during TB classical six-month treatment, particularly the enhancement of the CD4^+^ T cell responses in accelerating bacterial clearance using the mouse model. This strategy has been suggested before by many to be a possible way to reduce TB treatment duration (Costa et al., 2016; Costa et al., 2021). In fact, the CD4^+^ T cell response is known to be the cornerstone of TB immunity, and if this is combined with the anti-TB drugs, one would think that it will result in improved bacterial clearance and therefore reduced treatment duration (Maiga et al., 2012; Maiga et al., 2015; Costa et al., 2016). However, the perspective article showed that this is not sufficient to achieve the expected reduction when the authors used different strategies during the first months of the TB treatment intensive phase (first two months), including the infusion of IL-12, immunization ESAT-6 and Ag85B (TB specific peptides) and or adoptive transfer of Th1-specific CD4^+^ T cells to ESAT-6 or Ag85B. The authors recognize that the failure may have to do with the fact that the boosting took place when bacteria cells were actively dividing and thus when TB bacteriostatic antibiotics were the most performant. This strategy could still be useful in the later continuation phase of the treatment when antibiotics tend to be less efficient and when any immune support on slowly dividing bacterial cells could make a significant difference. This opens another avenue of research to be investigated extensively.

We believe that the manuscripts contained in this Research Topic bring important new data as well as thoughtful insights about how host-directed strategies can be developed and employed in the prevention, diagnosis and treatment of TB.

## Author Contributions

All authors contributed to the article and approved the submitted version.

## Conflict of Interest

The authors declare that the research was conducted in the absence of any commercial or financial relationships that could be construed as a potential conflict of interest.

## Publisher’s Note

All claims expressed in this article are solely those of the authors and do not necessarily represent those of their affiliated organizations, or those of the publisher, the editors and the reviewers. Any product that may be evaluated in this article, or claim that may be made by its manufacturer, is not guaranteed or endorsed by the publisher.
